# Oncogenic Gαq Signaling Remodels the Tumor Surfaceome and Rewires Intracellular Networks in Uveal Melanoma Models

**DOI:** 10.3390/cancers18121891

**Published:** 2026-06-10

**Authors:** Rakesh Mani, Leonie Enzinger, Chiara Thömmes, Daniel Devlitšarov, Alexander C. Rokohl, Christine Deisl, Ludwig M. Heindl, Jan Pruszak

**Affiliations:** 1 Institute of Anatomy and Cell Biology, Paracelsus Medical Private University (PMU), 5020 Salzburg, Austria; 2 Center for Anatomy and Cell Biology, Salzburg and Nuremberg, Paracelsus Medical Private University (PMU), 5020 Salzburg, Austria; 3 Department of Ophthalmology, Faculty of Medicine, University Hospital of Cologne, University of Cologne, 50937 Cologne, Germany

**Keywords:** cluster-of-differentiation (CD), surface antigen, network-based analysis, ocular oncology

## Abstract

Background: Dysregulated G protein-coupled receptor (GPCR) signaling is increasingly implicated as an important driver for oncogenesis. Uveal melanoma (UM) represents a highly metastatic intraocular malignancy primarily driven by activating mutations in G protein family members Gαq/11. Although Tebentafusp, the first FDA-approved bi-specific T-cell engager for UM, improves survival, its activity is restricted to specific human leukocyte antigen (HLA) alleles, highlighting the need to identify broadly expressed targetable proteins for immunotherapeutic strategies. Here we aimed to define surfaceome and phospho-signaling signatures associated with oncogenic Gαq-signaling. Methods: Heterologous and UM in vitro systems were used to interrogate Gαq-driven changes. HEK293T cells were transfected with wild-type Gαq or the oncogenic Gαq (R183Q) mutant, with surface marker profiles quantified by flow cytometry. Complementary immunophenotyping was performed in the Gαq-mutant UM cell line MP46 and Gα11-mutant line MP41. Kinase phosphorylation was assessed in control and Gαq mutant conditions followed by effect size estimation (Hedges’ g), Welch’s *t*-test, principal component analysis, and Spearman correlation-based network analysis of surface and phosphoprotein readouts. Results: Hyperactive Gαq in HEK293T cells induced graded remodeling of surface protein profiles, including reduced CD56 (NCAM) and CD49c (ITGA3) expression. Similarly, in UM models, MP46 versus MP41 had limited expression of CD56 and CD49c. Moreover, phospho kinase profiling and network analysis identified altered surface-phosphoprotein relationships, including a CD56-p70 S6 kinase association. Conclusions: These data provide new insights into Gαq-driven modulators of UM phenotype of relevance for studies of tumor–microenvironment interaction and metastasis.

## Simple Summary

Uveal melanoma is the most common eye cancer and frequently spreads to other organs, particularly the liver, where treatment options remain limited. Although recent immunotherapies have improved outcomes for some patients, their effectiveness is often restricted to individuals with specific genetic backgrounds, highlighting the need for additional therapeutic targets. In this study, we investigated how mutations in Gαq, one of the principal genetic drivers of uveal melanoma, influence proteins located on the cancer cell surface. Using laboratory cell models and uveal melanoma cell lines, we identified alterations in several surface proteins associated with mutant Gαq signaling. These findings suggest that aberrant Gαq activity may influence how tumor cells interact with their surrounding environment and regulate intracellular signaling pathways. While further validation in patient samples and functional studies is required, our results provide an initial map of Gαq-associated surface protein and signaling changes that may support the development of new therapeutic strategies.

## 1. Introduction

Uveal melanoma (UM) is a rare, aggressive intraocular malignancy arising from melanocytes within the uveal layers of the eye, which include the iris, ciliary body, and choroid [1]. Although primary tumors can often be effectively managed with local therapies, UM has a marked propensity for metastatic dissemination, most frequently to the liver. Once metastasis occurs, prognosis remains poor, with five-year survival rates declining to approximately 15% [2,3]. More than half of patients ultimately develop metastatic disease, and effective systemic therapies to prevent or control metastasis remain limited [4].

At the molecular level, UM is often characterized by activating mutations in the heterotrimeric G-protein α subunits Gαq or Gα11, encoded by the *GNAQ* and *GNA11* genes [5]. Under physiological conditions, GPCR activation induces GDP-GTP exchange on the Gα subunit, leading to transient and tightly regulated downstream signaling. In contrast, Gαq/11 mutations, most commonly occurring at codons R183 or Q209, result in constitutive activation of downstream pathways, such as MAPK, PLCβ, and YAP signaling, which contribute to tumor initiation and progression in UM [6].

Despite advances in defining the genetic drivers of UM, these insights have not translated into substantial improvements in clinical outcomes for patients with metastatic disease. Conventional chemotherapies and immune checkpoint inhibitors have shown limited efficacy [7,8,9]. Tebentafusp, a bispecific T cell receptor-based therapy and the first FDA-approved systemic treatment for UM, has shown a survival benefit [10,11]; however, its use is restricted to patients expressing the HLA-A*02:01 allele, limiting its applicability to a subset of patients [12]. This limitation highlights the need to identify additional, broadly expressed targetable proteins in UM that may be amenable to therapeutic intervention. In this context, surface markers represent an underexplored class of clinically actionable targets for immunotherapy in solid tumors such as UM [13,14,15]. Mapping the Gαq-driven surfaceome remodeling axis offers a systematic, unbiased approach to identify structurally exposed candidate markers that are altered downstream of primary oncogenic driver mutations.

Moreover, increasing evidence highlights the importance of tumor–microenvironment interactions, including those involving vascular, stromal, and extracellular matrix components, in regulating cancer progression, metastatic survival, and therapeutic resistance [16,17]. Surface markers are key mediators of these interactions, regulating processes such as cell adhesion, migration, immune engagement, and vascular association [18,19]. Furthermore, oncogenic signaling has been shown to remodel the composition of cell surface markers and influence tumor behavior [20]. However, the extent to which mutant Gαq signaling in UM contributes to surface marker remodeling remains insufficiently characterized.

In this study, we performed systematic profiling of cell surface marker expression across Gαq-driven model systems, including HEK293T cells expressing oncogenic Gαq and the uveal melanoma cell lines MP46 and MP41. By integrating surface marker profiling with intracellular signaling analyses, we aimed to identify Gαq-associated phenotypic signatures relevant to uveal melanoma biology. Importantly, the Gαq R183Q mutation investigated in this study affects a distinct regulatory region within the GTPase domain and is mechanistically separate from the more commonly studied Q209 hotspot mutation.

## 2. Materials and Methods

### 2.1. Cell Line and Culture Protocols

The human UM cell lines MP41 and MP46 (CRL-3297 and CRL-3298) were obtained from the American Type Culture Collection (ATCC, Manassas, VA, USA). Cells were maintained in RPMI-1640 medium (Gibco, Waltham, MA, USA) supplemented with 20% fetal bovine serum (FBS; PAN-Biotech GmbH, Aidenbach, Germany) and cultured at 37 °C in a humidified incubator with 5% CO_2_. Cells were routinely passaged and maintained at approximately 80% confluency. Human embryonic kidney cells (HEK293T; ACC-635) were obtained from the Leibniz-Institut DSMZ-Deutsche Sammlung von Mikroorganismen und Zellkulturen GmbH, Braunschweig, Germany, and cultured in DMEM/F12 medium (PAN-Biotech GmbH, Aidenbach, Germany) supplemented with 10% (*v*/*v*) FBS under the same incubation conditions (37 °C, 5% CO_2_).

### 2.2. Site-Directed Mutagenesis

The *GNAQ* c.548G>A point mutation, encoding the Gαq R183Q protein, was introduced into the wild-type Gαq mammalian expression vector (pCMV6-Entry, RC209199; Origene Technologies, Rockville, MD, USA) using site-directed mutagenesis. The quickchange site-directed mutagenesis kit (Agilent Technologies, Santa Clara, CA, USA) was employed according to the manufacturer’s instructions. Mutation-specific primers (Microsynth, Balgach, Switzerland) were designed: forward 5′-CAAGATGTGCTTAGAGTTCAAGTCCCCACCACAGGGATC-3′ and reverse 5′-GATCCCTGTGGTGGGGACTTGAACTCTAAGCACATCTTGT-3′. Successful incorporation of the c.548G>A mutation was confirmed by DNA sequencing.

### 2.3. Plasmid Transfection

HEK293T cells were seeded at a density of 3 × 10^5^ cells/cm^2^ in 6-well plates. After 24 h, cells were transfected with 3 µg of plasmid DNA encoding mutant Gαq using Turbofectin 8.0 (OriGene Technologies, Rockville, MD, USA) according to the manufacturer’s instructions. Control and wild-type cells were transfected with respective vector plasmids adjusted to molar equivalence with the mutant Gαq constructs, under identical experimental and transfection conditions. Transfection efficiency and protein overexpression were confirmed by immunoblotting.

### 2.4. Flow Cytometry

Cells were counted, and 1 × 10^5^ cells were aliquoted into each tube for control and experimental groups. Cells were then washed twice with flow buffer (PBS supplemented with 2% FBS) by centrifugation. Subsequently, cells were incubated with 0.5–1 µg/mL or a 1:50 dilution of fluorophore-conjugated primary antibodies for 1 h at room temperature on an orbital shaker. Details of all antibodies are provided in Supplementary Table S1. After incubation, cells were washed twice with flow buffer and resuspended in 200 µL of flow buffer for analysis. Flow cytometry was performed using a BD Accuri C6 (BD Biosciences, Franklin Lakes, NJ, USA), and data were analyzed with BD CFlow software (v1.0.227.4). Forward and side scatter parameters were used to gate for cell size and exclude doublets, as previously described (Supplementary Figure S1) [21,22]. Fluorescence gating was based on the respective fluorophore conjugated to the primary antibody. A minimum of 1 × 10^4^ gated events per sample were analyzed. All experiments were performed using three independent biological replicates, defined as independently cultured and transfected cell populations.

### 2.5. Immunoblotting

Cells were lysed using MPER^TM^ mammalian protein extraction reagent (Thermo Fisher Scientific, Waltham, MA, USA) supplemented with protease (Sigma-Aldrich, St. Louis, MO, USA) and phosphatase inhibitors (Thermo Fisher Scientific, Waltham, MA, USA). Protein concentrations were determined using the Bradford assay. Equal amounts of protein (10–20 µg) were mixed with 6× Laemmli buffer, heated at 65 °C for 10 min, and separated by SDS-PAGE (Bio-Rad, Hercules, CA, USA). Proteins were transferred to PVDF membranes (Merck KGaA, Darmstadt, Germany) using a wet transfer system. Membranes were blocked with 3% bovine serum albumin (Thermo Fisher Scientific, Waltham, MA, USA) in PBS containing 0.1% Tween-20 (AppliChem GmbH, Darmstadt, Germany) and incubated with HRP-conjugated antibodies (Supplementary Table S1). Signals were detected using enhanced chemiluminescence (Bio-Rad, Hercules, CA, USA).

### 2.6. Phospho-Kinase Profiling

Kinase phosphorylation was assessed using a commercially available phospho-kinase assay (R&D Systems, Minneapolis, MN, USA) following established protocols [23]. Proteins were extracted using RIPA lysis buffer supplemented with protease and phosphatase inhibitors, and protein concentrations were determined by the Bradford assay. Between 200 and 400 µg of total protein per sample was applied to the array. Signal detection was performed using a ChemiDoc imaging system, Image Lab Touch software v3.0.1.14 (Bio-Rad Laboratories, Hercules, CA, USA) (Supplementary Figure S2), and densitometric analysis of pixel intensity was carried out with Fiji software (v1.54k) to quantify relative phosphorylation levels. All experiments were performed using three independent biological replicates, defined as independently cultured and transfected cell populations.

### 2.7. Statistical Analysis

All statistical analyses were performed in Python (v3.13.9; Anaconda distribution, build 21 October 2025) using NumPy (v2.3.5), SciPy (v1.16.3), pandas (v2.3.3), scikit-learn (v1.7.2), matplotlib (v3.10.6), seaborn (v0.13.2), and NetworkX (v3.5). Delta percentage-positive values were calculated as the difference between the mean percentages of positive cells in the test condition minus the control condition. Group differences were quantified using effect sizes calculated as Hedges’ g, and statistical significance was assessed using two-tailed Welch’s *t*-tests. Hedges’ g was interpreted as small (0.2–0.5), medium (0.5–0.8), or large (>0.8) effect sizes accordingly. Volcano plots were generated by plotting Hedges’ g (effect size) on the *x*-axis versus −log_10_ (unadjusted *p*-value) from two-tailed Welch’s *t*-tests on the *y*-axis for each marker. For heatmap visualization, protein and surface marker values were Z-score normalized across rows to center each marker at zero and scale by its standard deviation. Principal component analysis (PCA) was performed on Z-score-normalized data using scikit-learn to summarize variance and visualize clustering of samples.

For integrative network analysis, bipartite networks were constructed between the top ten flow cytometry markers and the top ten phospho-proteins ranked by absolute Hedges’ g. Spearman rank correlations (ρ) were calculated separately for control and mutant samples (n = 3 biological triplicates per group). Differential correlation (Δρ = |ρ_control − ρ_mutant|) was used to quantify network rewiring. Statistical significance was assessed using an exact permutation test across all possible allocations of three samples per group (C(6,3) = 20 permutations), followed by Benjamini–Hochberg false discovery rate (FDR) correction across all hundred flow-phospho protein pairs. Network edges were retained using thresholds of |ρ| > 0.30 and permutation *p* < 0.45. Network layouts were generated using a force-directed spring algorithm with a fixed random seed to ensure reproducible node positioning.

Data visualization included Z-score-normalized heatmaps, volcano plots, PCA plots, delta plot summarizing mean shifts across conditions, and network visualizations.

## 3. Results

### 3.1. Generation of the GNAQ c.548G>A (Gαq R183Q) Mutant Construct

**Figure 1:**
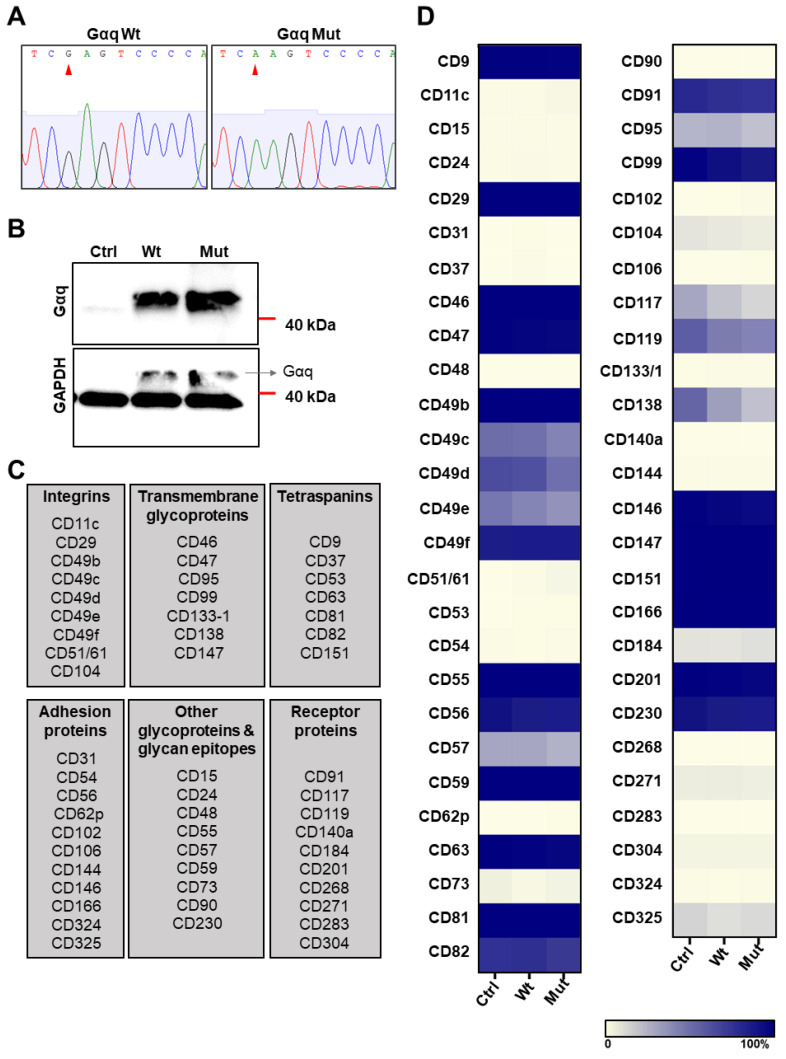
Generation of oncogenic Gαq R183Q mutant and surface marker profiling. (**A**) Sanger sequencing confirming the nucleotide substitution at position 548 (red arrow) corresponding to the R183Q mutation. (**B**) Representative Western blot of HEK293T cells transfected with Ctrl, Wt, or Mut constructs. GAPDH served as loading control. The uncropped blots are shown in Figure S3. (**C**) Functional classification of the analyzed surface markers. (**D**) Heatmap of mean percentage-positive cells for 53 CD markers across all conditions (n = 3 biological replicates); color intensity indicates marker positivity. Ctrl, control vector; Wt, Gαq wild-type; Mut, Gαq R183Q oncogenic mutant.

The oncogenic Gαq R183Q variant was generated by introducing a point mutation at c.548G>A in the wild-type *GNAQ* coding sequence, and successful incorporation of the mutation was confirmed by Sanger sequencing (Figure 1A). The mutant construct was subsequently transfected into HEK293T cells, and overexpression of the Gαq protein was verified by Western blot analysis (Figure 1B and Figure S3).

### 3.2. Surface Marker Profiling upon Oncogenic Gαq Expression

Surface marker expression was assessed following Gαq overexpression by screening a panel of more than fifty surface markers encompassing integrins, tetraspanins, immunoglobulin superfamily adhesion molecules, receptors, and glycoproteins (Figure 1C). Mean percentage-positive cells were visualized using a heatmap to provide an overview of surface marker expression across experimental conditions (Figure 1D). Median fluorescence intensity (MFI) values are provided in Supplementary Table S2.

**Figure 2:**
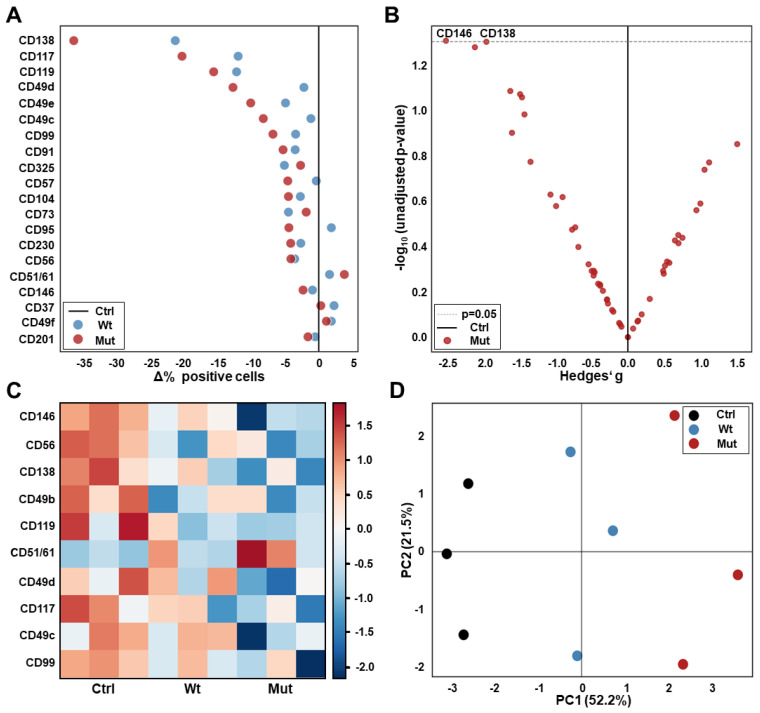
Oncogenic Gαq hyperactivation modulates the surface marker landscape. (**A**) Δ% positive cells for the top 20 surface markers across conditions (Wt or Mut versus Ctrl). (**B**) Volcano plot of surface markers comparing Mut versus Ctrl (Hedges’ g effect size versus –log_10_ *p*-value, two-tailed Welch’s *t*-test, unadjusted). (**C**) Heatmap of Z-score normalized expression for the top ten markers ranked by absolute Hedges’ g (>0.8, Mut versus Ctrl), showing individual biological replicates (n = 3 per condition). (**D**) PCA of the same top ten markers, summarizing variance and sample distribution across biological replicates. Ctrl, control vector; Wt, Gαq wild-type; Mut, Gαq R183Q oncogenic mutant.

Surface marker changes were quantified using delta percentage-positive cells for wild-type and mutant Gαq relative to control (Figure 2A). Both wild-type and mutant Gαq overexpression resulted in measurable shifts in surface marker expression compared with control cells, indicating that Gαq signaling influences the surface marker landscape. Notably, oncogenic Gαq expression was associated with more pronounced changes in surface protein expression patterns than wild-type Gαq.

### 3.3. Identification of Surface Markers with Large Effect Sizes upon Oncogenic Gαq Expression

The magnitude of surface marker changes was quantified using statistical significance (two-tailed Welch’s *t*-test) and effect sizes (Hedges’ g) for each marker comparing mutant versus control conditions and visualized using a volcano plot (Figure 2B). Two surface markers, CD138 and CD146, reached nominal statistical significance (*p* < 0.05). In addition to these, several surface markers displayed large effect sizes, including CD49b, CD49c, CD49d, CD51/61, CD56, CD99, CD117, and CD119, indicating shifts of potential biological relevance. The top ten markers ranked by absolute Hedges’ g were Z-score normalized and visualized as a heatmap across conditions (Figure 2C). This revealed a trend toward progressive divergence in surface marker expression from control to wild-type and further to mutant Gαq overexpression. PCA performed on these markers revealed a discernible shift in clustering between control and mutant samples (Figure 2D), consistent with differences in surface marker profiles between conditions.

### 3.4. Surface Marker Class-Based Composite Analysis

**Figure 3:**
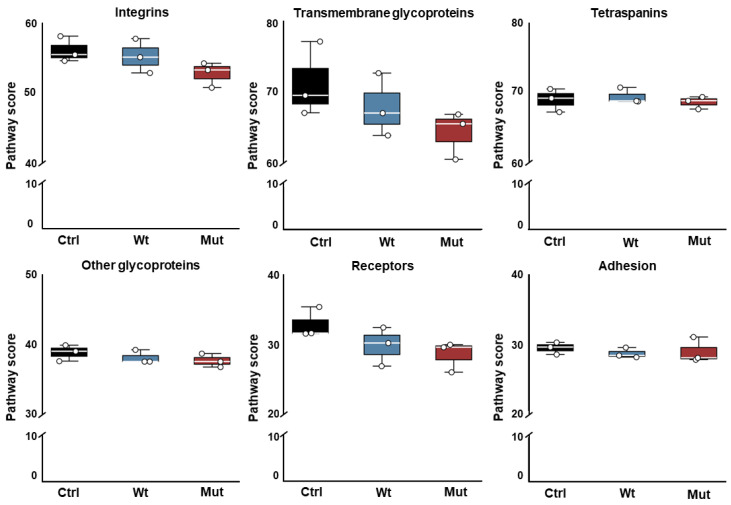
Composite analysis of surface marker classes upon oncogenic Gαq expression. Surface markers were grouped by functional class (see Figure 1C), and the composite score for each class is shown for Ctrl, Wt, and Mut conditions (n = 3 biological replicates per condition). Boxplots display medians and interquartile ranges, with individual data points overlaid. Class-level Hedges’ g effect sizes were: integrins, −1.454; transmembrane glycoproteins, −1.265; receptors, −1.587; tetraspanins, −0.226; adhesion molecules, −0.289; other glycoproteins, −0.882. A broken *y*-axis highlights class differences. Ctrl, control vector; Wt, Gαq wild-type; Mut, Gαq R183Q oncogenic mutant.

Surface markers were classified according to their functional categories, such as integrins, tetraspanins, adhesion molecules, receptors, and glycoprotein classes (see Figure 1C). For each category, composite scores were calculated by averaging the percentage-positive values of markers within each class, enabling comparison across experimental conditions. Integrins, glycoproteins, and receptor-associated surface marker clusters were modulated, as indicated by large Hedges’ g effect sizes, in cells expressing the mutant Gαq compared with control cells (Figure 3).

### 3.5. Surface Marker Expression in Uveal Melanoma Cell Lines

**Figure 4:**
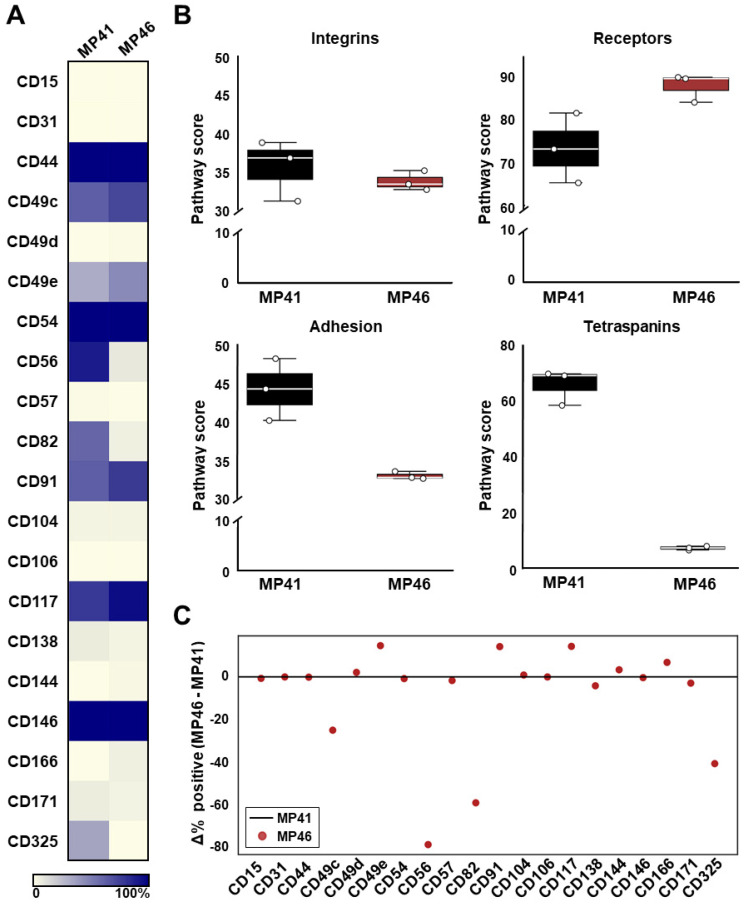
Differential surface marker expression in uveal melanoma cell lines. (**A**) Heatmap showing mean percentage-positive cells for 20 CD surface markers in MP41 and MP46 cells. Color intensity reflects marker positivity. (**B**) Composite analysis of surface marker classes, showing the composite score for each functional class. Boxplots display medians and interquartile ranges with individual data points overlaid. (**C**) Delta percentage-positive values (Δ% = MP46 − MP41) for each surface marker, highlighting relative differences between the two cell lines (n = 3 biological replicates per cell line).

Having identified mutation-associated surface marker modulation in the Gαq-driven heterologous HEK293T model system, complementary immunophenotypic patterns were studied in the two commonly used UM patient cell lines: MP46 cells, which harbor a hyperactive Gαq mutation, and MP41 cells, which carry a hyperactive Gα11 mutation [24,25]. Mean percentage-positive cells for a targeted panel of 20 surface markers, enriched for proteins involved in tumor–microenvironment interactions, were visualized using a heatmap (Figure 4A). Six of these markers were identified as modulated in the HEK293T oncogenic Gαq model system (see Figure 2C). The markers were further grouped into functional classes and composite scores were calculated. The adhesion molecules, tetraspanins, and receptor-associated surface marker classes were differentially expressed in MP46 cells compared with MP41 cells (Figure 4B).

Direct comparison using delta percentage-positive analysis identified four markers (CD49c, CD56, CD82, and CD325) with lower expression levels in MP46 cells relative to MP41 cells (Figure 4C). Importantly, CD49c and CD56 also showed reduced expression in HEK293T cells expressing mutant Gαq. Collectively, these findings indicate distinct surface marker profiles between the Gαq-mutant MP46 and the Gα11-mutant MP41 lines. Given that these are independent patient-derived tumors with broader genomic divergence, we interpret the lower CD49c and CD56 levels in MP46 as consistent with, but not definitive proof of, Gαq mutation-associated patterns suggested by the HEK293T model system.

### 3.6. Regulation of Intracellular Signaling upon Oncogenic Gαq Expression

**Figure 5:**
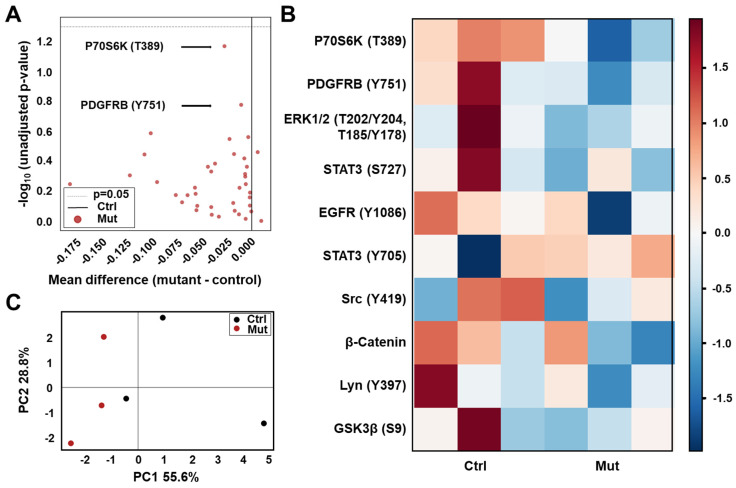
Oncogenic Gαq signaling modulates intracellular phospho-proteins. (**A**) Volcano plot showing phospho-protein differences between Mut and Ctrl in HEK293T cells. *X*-axis: mean difference; *Y*-axis: −log_10_ (*p*-value, unadjusted). Arrows indicate the top two proteins with the largest effect sizes. (**B**) Heatmap of Z-score normalized expression for the top ten phospho-proteins ranked by absolute Hedges’ g. Individual biological replicates are shown. (**C**) PCA of the same top ten phospho-proteins, summarizing variance and clustering across all replicates (n = 3 biological replicates). Ctrl, control vector; Mut, Gαq R183Q oncogenic mutant.

Given that surface markers involved in cell–cell interactions can influence intracellular signaling, alterations in phosphorylation pathways were investigated upon mutant Gαq expression using a phospho-kinase proteome profiler array. Mean differences between mutant and control conditions were calculated for each phospho-protein, and statistical significance was assessed using two-tailed Welch’s *t*-tests (unadjusted), and results visualized using a volcano plot (Figure 5A).

Effect size (Hedges’ g)-based top ten modulated phospho-proteins were Z-score normalized to enable comparative visualization across samples, and this suggested progressive differences between control and mutant conditions (Figure 5B). PCA based on these phospho-proteins indicates modest separation between control and mutant samples (Figure 5C).

### 3.7. Surface-Phospho Protein Correlation Analysis upon Oncogenic Gαq Expression

**Figure 6:**
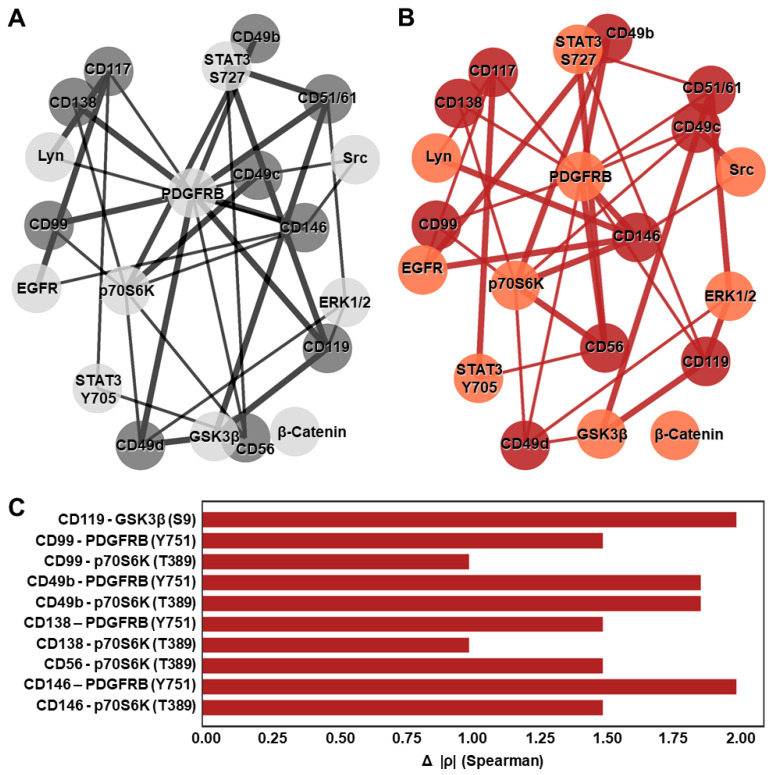
Network analysis reveals rewired surface-phospho signaling upon oncogenic Gαq overexpression. (**A**,**B**) Bipartite networks of surface markers and phospho-proteins. Nodes represent proteins; edges indicate Spearman correlations (|ρ| > 0.3). (**C**) Top ten most rewired surface-phospho interactions (FDR-corrected), highlighting mutation-dependent changes. Control (Ctrl): light grey, phosphoproteins; dark grey, surface proteins. Mutant (Mut, Gαq R183Q oncogenic variant): orange, phosphoproteins; firebrick, surface proteins.

Changes in the interaction between cell surface markers and intracellular signaling pathways upon oncogenic Gαq expression were determined via a targeted Spearman correlation-based network analysis. The top ten surface markers and top ten phospho-proteins identified earlier (see Figure 2C and Figure 5B) were used to construct a rewiring matrix (Δρ = |ρ_control − ρ_mutant|) to quantify mutation-dependent changes in correlation strength between conditions. Bipartite network analysis revealed differences in connectivity patterns between control and mutant states (Figure 6A,B), suggesting changes in surface-to-intracellular signaling relationships. The top ten rewired interaction pairs in the mutant condition further highlighted changes in associations between specific surface markers and downstream phospho-effectors (Figure 6C). Collectively, these findings support a model in which oncogenic Gαq signaling can reshape the surface-to-intracellular communication networks.

## 4. Discussion

Effective therapeutic strategies to prevent or limit metastatic progression in UM remain limited [12]. To address this unmet clinical need, we systematically characterized surface marker signatures in UM and Gαq-driven heterologous cell models. This study builds on prior evidence across various cancer types that surface markers can serve as actionable immunotherapy targets [13,14,18,26], such as SEMA4 in multiple myeloma [15,27], and by extensive literature linking surface protein remodeling to metastatic progression [21,23,28,29]. Our results indicate that hyperactive Gαq signaling is associated with coordinated modulation of multiple surface markers along with corresponding changes in intracellular kinase cascades, which together may influence tumor-vascular and extracellular matrix interactions. These findings suggest that oncogenic Gαq may function not only as an intracellular signaling driver but also as a modulator of surface proteins.

### 4.1. Oncogenic Gαq Signaling Modulates Surface Marker Landscapes

Previous studies have demonstrated that oncogenic signaling pathways can remodel the tumor cell surface, weakening adhesion and promoting metastatic dissemination [20,30]. Consistent with this, our data indicate that hyperactive Gαq expression is associated with coordinated alterations in surface markers involved in cell-cell adhesion and tumor-vascular interactions [31,32,33]. Notably, adhesion-associated markers, such as CD56 (NCAM) and CD49c (α3 integrin, ITGA3), which were downregulated in HEK293T cells expressing mutant Gαq, also had low expression in MP46 cells. Reduced expression of these adhesion molecules has been reported in other cancer types to weaken tissue anchorage and promote migratory behavior [34,35,36]. Collectively, these findings support a model in which oncogenic Gαq signaling contributes to surface marker remodeling, potentially influencing cell–cell interactions. Moreover, inclusion of wild-type Gαq aids in distinguishing the mutation-specific effects from generalized overexpression. The top 10 surface markers, including CD56 and CD49c, exhibited graded changes from control to wild-type to mutant conditions, suggesting that oncogenic hyperactivation might amplify surface remodeling beyond basal Gαq overexpression.

### 4.2. Network Analysis Reveals Rewired Surface-Phospho Signaling upon Oncogenic Gαq Expression

Tumor cell surface markers dynamically interact with the microenvironment and can influence intracellular kinase signaling [37,38]. To investigate how oncogenic Gαq-driven surface remodeling may impact these pathways, we analyzed intracellular phospho-signaling networks at the protein level. Mutant Gαq signaling induced broad, coordinated changes across multiple kinase pathways rather than activation of a single dominant axis, consistent with its pleiotropic role as a GPCR-associated mediator [39,40,41,42,43]. Many of the phospho-proteins tested are part of pathways previously implicated in cancer progression [44,45,46,47].

Integration of surface marker expression with phospho-protein data using Spearman correlation-based network analysis highlights putative altered connectivity patterns in Gαq mutant-expressing cells. This included changes in correlation-based relationships between surface markers and downstream kinases, such as CD56-p70 S6 kinase, CD119-GSK3β, and CD146-PDGFRβ/p70 S6 kinase. These findings indicate that constitutive Gαq activation may be associated with coordinated changes in both surface phenotypes and intracellular signaling networks. Importantly, several of the interactions have previously described biological roles: CD146-PDGFRβ signaling has been associated with pericyte recruitment and GSK3β has been reported to regulate ubiquitination of the CD119 receptor, influencing its proteasomal degradation [48,49]. The presence of these known regulatory relationships supports the biological plausibility of the inferred correlations.

### 4.3. Limitations and Future Directions

Although this study provides a detailed characterization of Gαq-associated surface marker and phospho-signaling patterns, the majority of data were derived from in vitro models using HEK293T cells and a limited panel of UM cell lines. HEK293T cells were employed to examine the direct effects of hyperactive Gαq signaling independent of patient-specific variability. Nevertheless, HEK293T cells represent a non-melanoma, non-ocular cellular context and therefore cannot fully recapitulate the lineage-specific and microenvironmental signaling landscape of primary uveal melanoma. Accordingly, findings derived from the HEK293T system should be interpreted as a reductionist heterologous model of Gαq. Future studies using patient-derived samples and functional assays will be essential to validate the translational relevance of these observations. Additionally MP41 and MP46 cell lines represent non-isogenic, patient-derived cell lines; differential expression patterns cannot be attributed solely to Gαq versus Gα11 signaling and likely also reflect broader inter-tumoral heterogeneity. Given the limited sample size, statistical significance was interpreted cautiously, and effect size was used to prioritize biologically meaningful trends. This approach is further supported by the dynamic regulation of cell surface markers, which can undergo rapid remodeling through processes such as endocytosis [50], contributing to variability across biological replicates. Furthermore, the network analysis was intended strictly as an exploratory, hypothesis-generating visualization under small-sample constraints, where all statistical outputs should be interpreted cautiously and not as stable estimates of network topology. For example, the previously unreported CD56-p70 S6 kinase association observed here should be validated for cell migration/invasion, as well as immune co-culture assays as a necessary next step.

## 5. Conclusions

Our findings provide a framework for understanding how oncogenic Gαq signaling can modulate UM phenotypes associated with metastatic potential and highlights candidates of surface-accessible proteins as potential biomarkers or therapeutic targets. More broadly, the integrative surface-phospho network described here may yield novel testable hypothesis for mutation-informed UM studies and eventually developing novel treatment strategies.

## Data Availability

Dataset available on reasonable request from the authors.

## Supplementary Materials

The following supporting information can be downloaded at: https://www.mdpi.com/article/10.3390/cancers18121891/s1, Figure S1: Flow cytometry gating strategy and representative surface marker analysis. (A) Initial gating of the main cell population (P1) using forward scatter area (FSC-A) versus side scatter area (SSC-A) to exclude debris. (B) Doublet discrimination within P1 using FSC-A versus forward scatter height (FSC-H) to select single cells (P2) for analysis. (C) Representative flow cytometry plots gated on P2 within P1, showing surface expression of CD138 in HEK293T cells transfected with Ctrl, Wt, or Mut. Ctrl, control vector; Wt, Gαq wild-type overexpression; Mut, Gαq R183Q oncogenic mutant. Figure S2: Phosphoproteomic profiling and visualization of phospho-kinase array data. (A) Representative phospho-kinase membrane blot showing Ctrl and Mut overexpression in HEK293T cells. Blots were detected using streptavidin-HRP and chemiluminescence. (B) Transparent overlay of membrane images aligned with reference spots in the corners of each array to facilitate identification of individual phosphoproteins. (C) Coordinate map of the human phospho-kinase array indicating the location of each kinase corresponding to the array spots. Ctrl, control vector; Mut, Gαq R183Q oncogenic mutant. Figure S3: Raw Western blot data corresponding to Figure 1B. (A) Stain-free blot showing molecular weight markers and equal protein loading across samples. (B) Western blot for Gαq expression. (C) Corresponding GAPDH loading control. (D) Densitometric analysis of Western blot bands showing relative protein expression normalized to GAPDH. Error bar represents mean ± standard deviation (SD) of three independent biological replicates. Table S1: List of antibodies used in this study. Table S2: Median fluorescence intensity (MFI) values of surface markers analyzed across experimental conditions.
