# Supplementary material for: Oncogenic Gαq Signaling Remodels the Tumor Surfaceome and Rewires Intracellular Networks in Uveal Melanoma Models
Source: Cancers (Basel). 2026 Jun 10;18(12):1891. doi: 10.3390/cancers18121891 (PMC13296695; doi:10.3390/cancers18121891)
Supplement: Supplementary file 1 [file cancers-18-01891-s001.zip › Supp Table S1.pdf]

Supplementary Table S1. List of antibodies used in this study.

Antibody	Conjugate	Catalog no.	Vendor	µg ml-1
CD9	FITC	11-0098-42	eBioscience	1
CD11c	FITC	11-0116-41	eBioscience	1
CD15	FITC	11-0159-42	eBioscience	0.5
CD24	PE	12-0247-42	eBioscience	1
CD29	APC	17-0299-42	eBioscience	1
CD31	APC	303116	Biozym	0.8
CD46	PE	12-0469-42	eBioscience	1
CD47	FITC	11-0478-41	eBioscience	1
CD48	FITC	336705	Biolegend	1
CD49b	APC	17-0500-42	eBioscience	0.5
CD49c	APC	17-0494-42	eBioscience	0.5
CD49d	PE	12-0499-42	eBioscience	0.5
CD49e	AF488	53-0496-42	eBioscience	0.5
CD49f	APC	17-0495-82	eBioscience	1
CD51-61	FITC	11-0519-42	eBioscience	1
CD54	PE	12-0549-42	eBioscience	1
CD55	APC	311311	Biozym	1
CD56	PE	12-0567-42	eBioscience	0.5
CD57	FITC	11-0577-42	eBioscience	1
CD59	PE	12-0596-42	eBioscience	0.5
CD62p	PE	12-0628-41	eBioscience	0.5
CD63	PE	12-0639-42	eBioscience	0.5
CD73	PE	12-0739-42	eBioscience	0.5
CD81	APC	17-0819-42	Fisher	1
CD90	APC	17-0909-42	eBioscience	1
CD95	FITC	11-0959-41	eBioscience	1
CD99	FITC	371303	BioLegend	1
CD102	FITC	328507	Biozym	2
CD104	AF660	50-1049-82	eBioscience	1
CD106	PE	12-1069-42	eBioscience	0.5
CD117	APC	17-1178-42	eBioscience	1
CD119	PE	12-1199-41	eBioscience	1
CD138	APC	17-1389-42	eBioscience	0.5
CD140a	APC	MA1-10097	Fisher	0.5
CD146	APC	361015	Biozym	1
CD147	PE	12-1472-42	eBioscience	1
CD151	APC	17-1519-42	eBioscience	0.5
CD166	PE	12-1668-42	eBioscience	0.3
CD201	APC	351905	Biozym	1
CD230	PE	12-9230-42	eBioscience	0.5
CD271	AF647	560326	BD Biosciences	1
CD324	AF660	50-3249-82	eBioscience	1
CD325	APC	17-3259-42	eBioscience	1

Antibody	Conjugate	Catalog no.	Vendor	DF
CD37	APC	17-0379-41	eBioscience	1:50
CD53	PE	130-101-782	Miltenyi Biotec	1:50
CD82	PE	130-101-306	Miltenyi Biotec	1:50
CD91	PE	12-0919-41	eBioscience	1:50
CD133-1	APC	130-090-826	Miltenyi Biotec	1:50
CD144	FITC	BMS158FI	Labome	1:50
CD184	PE	12-9999-42	eBioscience	1:50
CD268	PE	12-9117-41	12-9117-41	1:50
CD283	FITC	130-100-000	Miltenyi Biotec	1:50
CD304	PE	130-090-533	BD Biosciences	1:50
