# Supplementary material for: Oncogenic Gαq Signaling Remodels the Tumor Surfaceome and Rewires Intracellular Networks in Uveal Melanoma Models
Source: Cancers (Basel). 2026 Jun 10;18(12):1891. doi: 10.3390/cancers18121891 (PMC13296695; doi:10.3390/cancers18121891)
Supplement: Supplementary file 1 [file cancers-18-01891-s001.zip › Supp Table S2.pdf]

Supplementary Table S2. Median fluorescence intensity (MFI) values of surface markers analyzed across experimental conditions.

Markers	Ctrl Mean	Ctrl SD	Wt Mean	Wt SD	Mut Mean	Mut SD
CD9	43,261.33	12625.26	45,274.67	11866.83	45,387.50	8429.213
CD11c	18,974.83	5409.758	21,103.67	7203.199	20,353.50	5581.56
CD15	12,835.33	4921.106	13,865.67	6909.634	12,201.67	3910.892
CD24	4,405.33	840.7689	4,101.17	695.1188	4,423.00	207.067
CD29	85,564.00	23287.11	85,880.33	23441.38	86,527.50	19486.73
CD31	3,923.00	945.0397	5,527.83	2656.652	3,738.00	919.1685
CD37	3,910.33	2406.429	2,852.67	378.0639	2,827.50	686.4141
CD46	34,840.67	7266.2	36,015.67	6238.51	34,603.17	4353.228
CD47	18,510.67	6078.376	17,561.33	6709.079	17,221.17	5666.495
CD48	14,480.17	3867.188	14,548.50	4582.117	14,619.83	3174.531
CD49b	19,123.67	650.8714	17,612.67	634.5332	16,845.67	737.4051
CD49c	3,729.00	558.2723	3,767.33	548.5493	3,546.83	547.2441
CD49d	9,741.83	866.3972	9,512.00	680.5782	8,957.17	836.6266
CD49e	16,779.67	1772.022	16,693.00	1399.585	16,518.00	1342.88
CD49f	5,293.33	2090.162	4,974.00	1729.023	5,136.33	1397.479
CD51/61	6,595.67	802.657	6,809.17	1105.172	6,940.00	1148.096
CD53	4,544.00	2272.805	4,089.17	1408.79	4,184.00	1466.083
CD54	6,532.17	2778.13	6,545.33	2495.561	6,462.17	2276.94
CD55	56,182.33	7391.672	45,180.67	7904.89	42,166.00	13300.38
CD56	14,597.17	828.6541	14,204.17	892.1071	13,743.33	613.5359
CD57	20,081.67	7035.276	20,397.50	7798.717	18,999.17	6119.25
CD59	129,872.33	25811.19	132,443.50	32390.67	133,895.33	22128.93
CD62p	5,897.00	2276.574	5,779.33	1894.033	5,870.67	2275.385
CD63	25,587.33	2227.565	27,120.00	5436.306	32,385.00	4270.665
CD73	5,142.50	1527.937	4,060.83	1252.101	5,364.00	717.2956
CD81	283,565.17	93616.67	295,807.00	60978.5	253,365.00	36676.37
CD82	6,994.17	1004.794	7,516.33	815.8636	7,029.33	778.6015
CD90	2,748.00	496.8219	2,856.00	393.9962	2,748.67	471.6846
CD91	8,703.67	1850.597	7,341.50	1531.839	7,467.17	995.855
CD95	12,168.17	3818.79	12,548.17	4262.524	12,279.50	4371.052
CD99	24,552.50	6148.377	18,932.83	4744.173	18,453.17	6665.858
CD102	15,808.67	1664.675	15,505.83	946.1298	15,197.67	1362.85
CD104	4,569.50	581.0759	4,373.17	892.5002	4,292.67	793.6954
CD106	6,357.50	493.2228	6,292.50	622.6181	6,271.00	657.2762
CD117	3,098.00	325.4704	2,987.83	271.5678	2,885.67	287.6549
CD119	8,013.00	479.5696	7,934.50	303.8013	7,810.50	526.2274
CD133-1	2,160.50	394.6875	2,189.00	266.9963	2,202.67	371.5176
CD138	4,331.33	356.8646	3,693.33	248.6008	3,487.50	174.8692
CD140a	2,264.00	543.011	2,231.00	336.6705	2,362.17	622.9876
CD144	20,551.33	2727.674	19,524.33	3425.718	17,671.17	3271.777
CD146	14,010.67	792.2869	11,631.00	871.1699	10,642.50	1060.616
CD147	485,487.67	90909.94	498,535.33	151310.6	535,372.33	86864.47
CD151	55,448.17	7501.295	58,814.17	10545.47	53,241.33	6198.849

CD166	19,527.17	4374.873	20,370.50	5770.885	21,962.17	8048.288
CD184	7,612.33	1533.636	7,408.50	1512.804	7,363.17	1387.333
CD201	52,489.33	12531.65	41,916.00	10302.39	34,827.50	11700.33
CD230	14,614.00	1789.045	14,212.00	1633.754	13,957.33	1373.363
CD268	5,182.00	1254.483	5,462.67	1306.772	5,341.00	1584.5
CD271	3,046.33	546.3793	3,051.83	469.8846	2,980.00	494.5736
CD283	11,705.67	6065.197	12,088.83	5467.38	11,728.17	4575.498
CD304	5,700.50	2287.803	6,108.17	2606.079	6,359.83	2088.57
CD324	4,163.33	1299.148	4,389.50	1566.111	4,436.00	1272.301
CD325	1,973.67	146.3227	1,965.17	115.8257	2,012.00	151.5949
