# Supplementary figures and images for: Oncogenic Gαq Signaling Remodels the Tumor Surfaceome and Rewires Intracellular Networks in Uveal Melanoma Models
Source: Cancers (Basel). 2026 Jun 10;18(12):1891. doi: 10.3390/cancers18121891 (PMC13296695; doi:10.3390/cancers18121891)

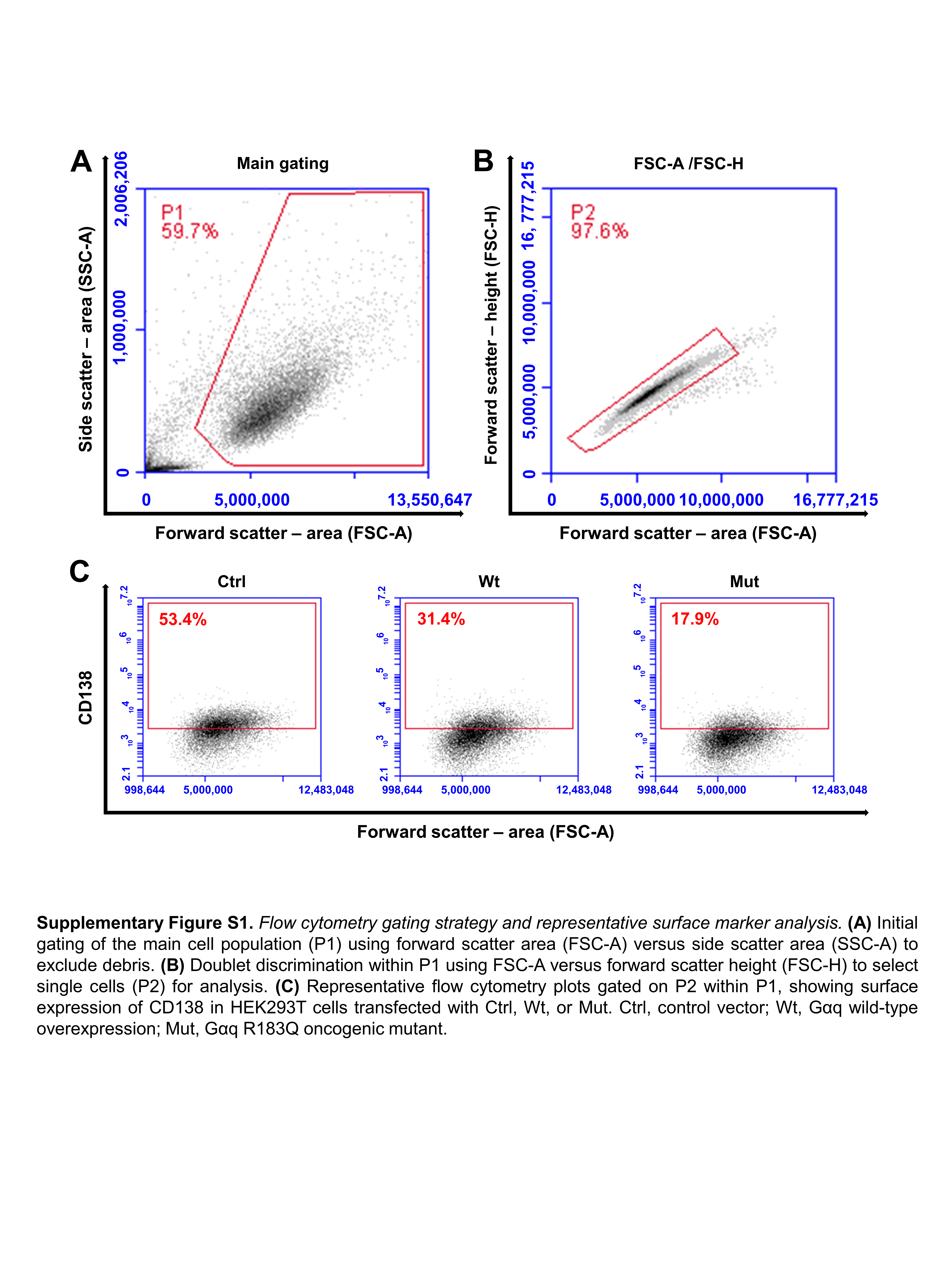

Supplement: Supplementary file 1 [file cancers-18-01891-s001.zip › Figure S1.tif]

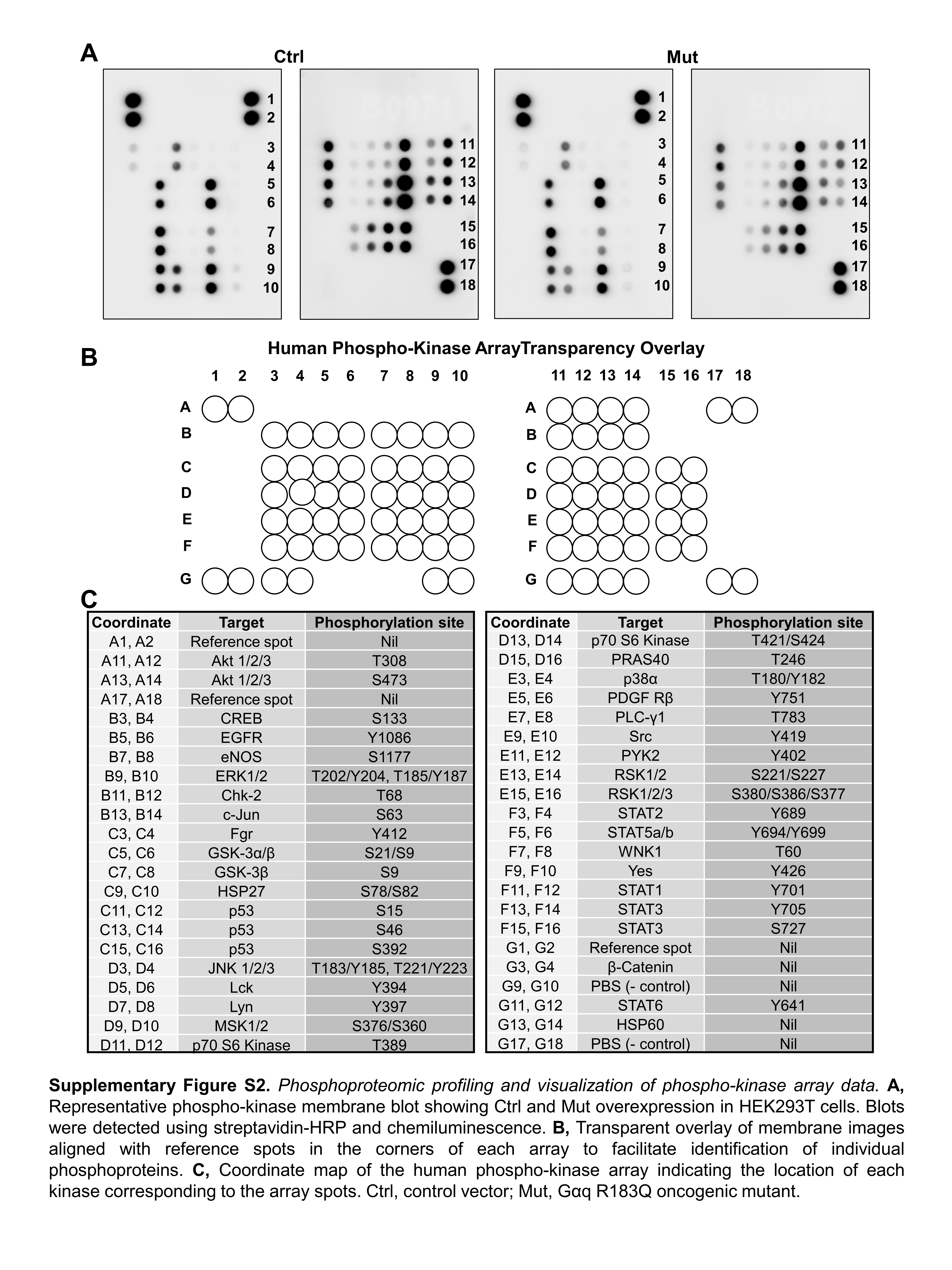

Supplement: Supplementary file 1 [file cancers-18-01891-s001.zip › Figure S2.tif]

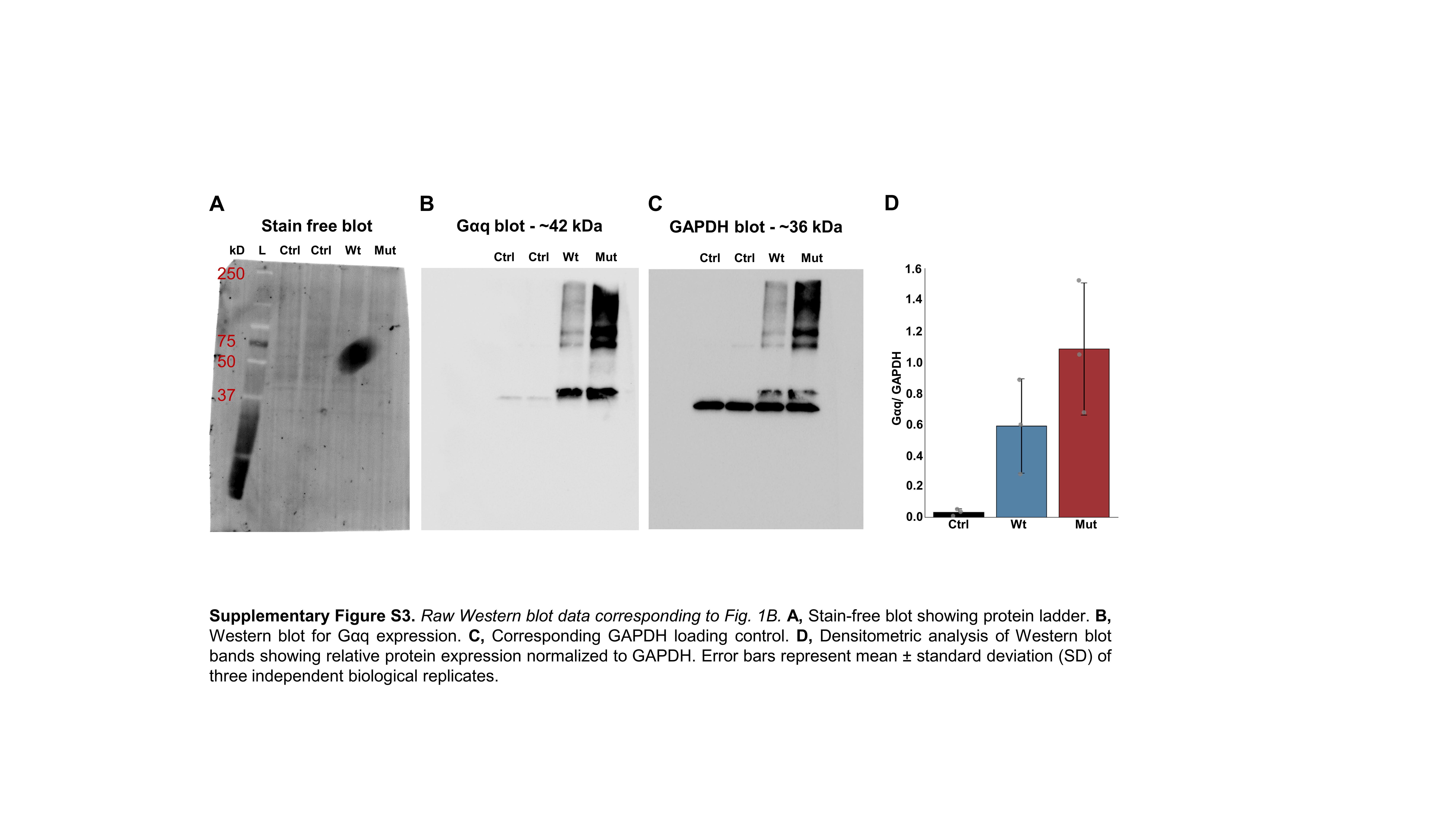

Supplement: Supplementary file 1 [file cancers-18-01891-s001.zip › Figure S3.tif]
